# Small‐bites versus large‐bites closure of midline laparotomies: A systematic review and meta‐analysis

**DOI:** 10.1111/codi.70073

**Published:** 2025-03-24

**Authors:** Stefan Morarasu, Sorinel Lunca, Luke O'Brien, Paul Lynch, Ana Maria Musina, Cristian Ene Roata, Raluca Zaharia, Wee Liam Ong, Gabriel‐Mihail Dimofte, Cillian Clancy

**Affiliations:** ^1^ 2nd Department of Surgical Oncology Regional Institute of Oncology (IRO) Iasi Romania; ^2^ Grigore T Popa University of Medicine and Pharmacy Iasi Iasi Romania; ^3^ Department of Colorectal Surgery Tallaght University Hospital Dublin 24 Ireland

**Keywords:** abdominal closure, incisional hernia, laparotomy, large bites, small bites

## Abstract

**Aim:**

Surgical site infection (SSI) and incisional hernia (IH) are common complications following midline laparotomy. The small‐bites technique for closing a midline laparotomy has been suggested to improve SSI and IH rates compared with the classic mass closure. The aim of this work was to perform a systematic review, meta‐analysis and fragility assessment of existing evidence comparing small‐bites and conventional closure.

**Method:**

The study was registered with PROSPERO. A systematic search of PubMed and EMBASE databases was performed for all comparative studies examining small‐bites versus conventional closure for midline laparotomy. The fragility index for randomized controlled trials (RCTs) was assessed and the number of outcomes required to render results insignificant using the Fisher exact test was calculated.

**Results:**

Seven studies were included, with a total of 3807 patients. Small bites was performed in 1768 and large bites in 2039. Follow‐up ranged from 12 to 52 months. On meta‐analysis of all studies, small bites is associated with a lower risk of IH (*p* < 0.00001), SSI (*p* = 0.0002) and wound dehiscence (*p* = 0.02). On meta‐analysis of RCTs there is a lower risk of IH (*p* = 0.01) but no difference in SSI (*p* = 0.06) or wound dehiscence (*p* = 0.73). Fragility is evident among RCTs reporting differences in IH rates.

**Conclusion:**

There is evidence to suggest that small‐bites closure provides a decreased likelihood of IH over varying follow‐up in RCTs but significant fragility exists among studies.

**What does this paper add to the literature?**

This is the first meta‐analysis to compare outcomes of the small‐bites versus large‐bites technique in terms of patient risk factors and incidence of surgical site infection, wound dehiscence and incisional hernia, with an examination of fragility of randomized studies showing that small bites is noninferior; however, there is significant fragility among randomized controlled trials.

## INTRODUCTION

Surgical site infection (SSI) and incisional hernia (IH) are among the most common complications following midline laparotomy and a major factor contributing to longer hospital stay, readmission and increased costs related to surgical care [1, 2, 3]. Continuous closure with slowly absorbable sutures is the current standard, and has been suggested to reduce the incidence of SSI and IH [4]. Techniques to further improve the closure technique, including optimal suture length and the use of small‐bites closure together with a lower‐diameter suture material, have not been universally adopted despite evidence [5].

Several studies have suggested the optimal method of midline laparotomy closure should include a suture to wound length ratio of 4:1 and the use of a 2.0 Polydioxanone (PDS) or slowly absorbable suture material with 5 mm bites of the anterior fascia spaced 5 mm apart [3, 4]. The use of this technique is suggested to produce a higher burst strength due to equal distribution of tension and less tearing of tissues compared with widely spaced high‐tension bites [6, 7, 8]. Experimental studies have shown that in mass closure sutures cut through the soft tissue such as muscle, peritoneum or subcutaneous fat when the abdomen is approximated, increasing the amount of necrotic tissue and thus favouring infection, which in turn has an association with the development of IH [9]. More so, other studies have shown that the tensile strength seems to be higher when sutures are placed 5 mm apart compared with at least 10 mm [6].

Despite showing better results in individual studies and being included in the European Hernia Society recommendations for midline laparotomy closure [3], the small‐bites technique is yet to be implemented by the majority of surgeons in their clinical practice [7]. There are concerns regarding the quality of evidence behind small bites, with only two major randomized clinical trials being done (ESTOIH [8] and STITCH [9]). While the STITCH trial [9] showed favourable evidence for small bites, the results of the ESTOIH trial [8] were equivocal. This systematic review and meta‐analysis aims to assess all current comparative robust evidence regarding the small‐bites technique versus mass closure including a critical analysis of patient factors in each group. In addition, the robustness of randomized controlled trials (RCTs) reporting the use of small bites is assessed to further quantify the quality of existing evidence.

## METHOD

### Literature search and study selection

**FIGURE 1:**
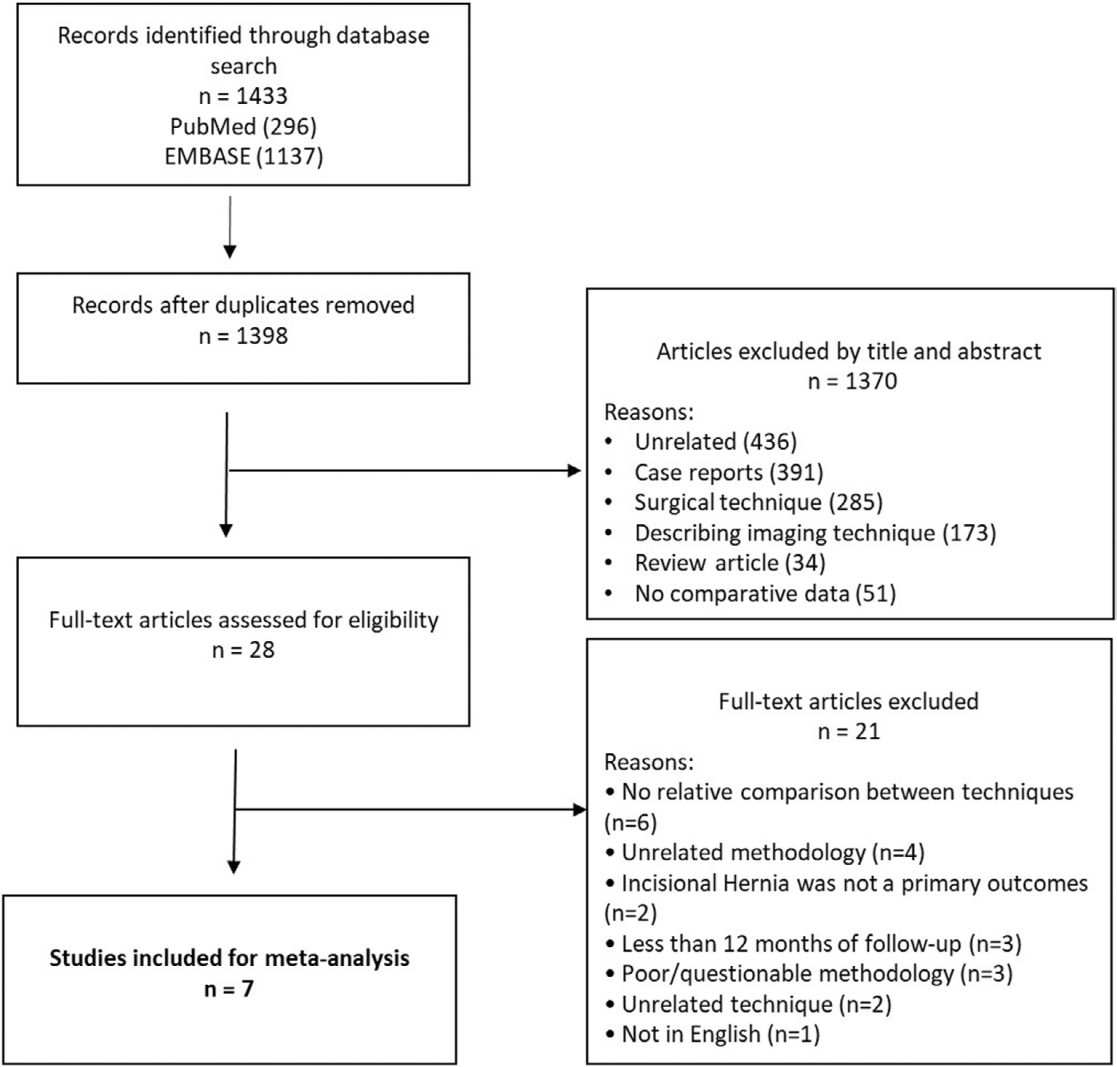
Preferred reporting items in systematic reviews and meta‐analysis (PRISMA) diagram.

**FIGURE 2:**
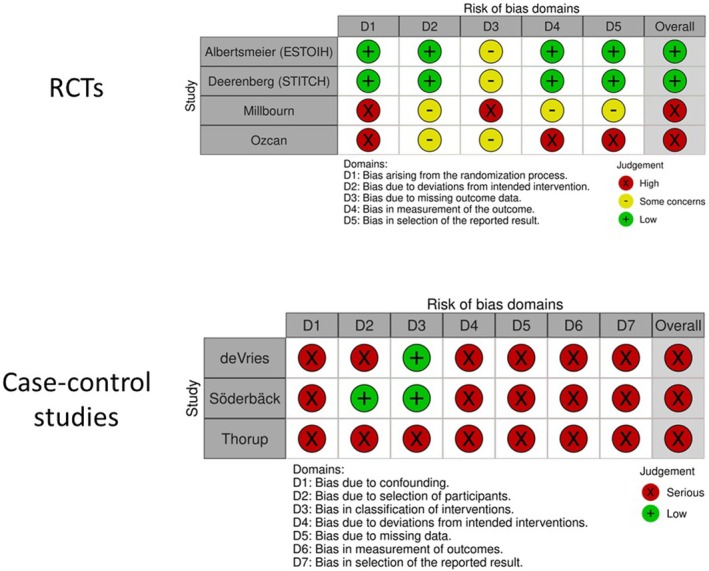
ROB2 (top) and ROBINS‐I (bottom) risk of bias assessment. Assessment of risk of bias was done by two authors (SM and CC). Randomized controlled trials (RCTs) were analysed via the ROB2 tool (top) while case–control studies were analysed with the ROBINS I tool (bottom). Each study was classified as having low/moderate/serious risk for each of the five or seven domains. Disagreements were resolved via consensus.

The study was registered with PROSPERO (International Prospective Register of Systematic Reviews). The study ID is CRD42023443519. A systematic search of PubMed and EMBASE databases was performed for all comparative studies examining surgical outcomes in patients who underwent small‐bites versus large‐bites closure of a midline laparotomy. The following search algorithm was used: (small OR short) AND (bites OR suture OR stitch) AND (laparotomy). Preferred Reporting Items for Systematic Reviews and Meta‐Analyses (PRISMA) guidelines [10] were used as the search protocol and the PRISMA checklist was followed to conduct the methodology (Figure 1). Inclusion criteria were used according to the Problem, Intervention, Comparison and Outcome (PICO) formula. The latest search was performed on 15 November 2024. Two authors (SM and LOB) assessed the titles and abstracts of studies found in the search and the full texts of potentially eligible trials were reviewed. Disagreements were resolved by consensus‐based discussion. The ROB2 and ROBINS‐I tools [11] (Figure 2) were used to quantify the quality of eligible studies, as previously done [12, 13, 14]. The references of the reviewed full texts were further screened for additional eligible studies. The corresponding author was contacted to clarify data extraction if additional information was necessary.

### Eligibility criteria

Studies written in English including comparative surgical data between small bites versus large bites were assessed for eligibility. After initial triage, studies with poor methodology and a high risk of bias were excluded from the final analysis. The primary endpoints were the rates of SSI, wound dehiscence and IH. Secondary endpoints included patient characteristics, suture length, duration of abdominal closure and length of stay. Studies including additional techniques for abdominal closure were excluded (i.e. mesh, tissue adhesive). Studies without comparative data were not included. Studies in which small bites was not the only intervention in the study group (SG) were excluded. Studies in which follow‐up for IH was less than 1 year were also excluded [15, 16, 17, 18], as were studies that did not report IH rates [15, 17, 19]. Retrospective studies that did not report on the patient characteristics between the two groups were excluded due to the unacceptable risk of selection bias.

### Data extraction and outcomes

For each eligible study the following data were recorded: authors’ names, journal, year of publication, study type, total number of patients and number of patients included in each group, small‐bites technique, large‐bites technique, type of suture used, mean age, mean body mass index (BMI), smoking status, cardiovascular comorbidities, respiratory comorbidities, diabetes, steroid use, previous laparotomy, American Society of Anesthesiologists (ASA) score, neoadjuvant therapy, type of surgery, suture to wound length ratio, number of sutures, suture length, time of closure, length of stay, SSI (superficial, deep), Clavien–Dindo classification, wound dehiscence, IH. For each study, the outcomes of interest were extracted and grouped into three main categories which were further analysed: (i) preoperative risk factors (male gender, smoking status, ASA grade, previous laparotomy, indication of surgery, type of procedure); (ii) intraoperative outcomes (wound length, duration of closure, suture length, suture to wound length ratio); (iii) postoperative outcomes (SSI, wound dehiscence, IH). The main outcomes were SSI, wound dehiscence and IH.

### Statistical analysis

Random‐effects models were used to measure all pooled outcomes as described by Der Simonian and Laird [20] and the OR was estimated with its variance and 95% CI. The random‐effects analysis weighed the natural logarithm of each study's OR by the inverse of its variance plus an estimate of the between‐study variance in the presence of between‐study heterogeneity. Each study is shown by the point estimate of the OR/mean difference (square proportional to the weight of each study) and 95% CI for the OR (extending lines); the combined ORs/mean difference and 95% CIs by random effects calculations are shown in the figures by diamonds.

As described previously [19], heterogeneity between ORs for the same outcome between different studies was assessed using the *I* ^2^ inconsistency test and chi‐square‐based Cochran's *Q* statistic test^20^ in which *p* < 0.05 is taken to indicate the presence of significant heterogeneity. To measure the robustness of the results of RCTs the fragility index (FI) [21, 22, 23] was calculated for each primary outcome. Data from each trial are presented in a two‐by‐two contingency table, and the FI was calculated in the manner described by Walsh et al. [22]. For time‐to‐event outcomes, the total number of events in each group over the entire follow‐up time was included. Events were added to the smaller event group and nonevents were simultaneously subtracted, while maintaining a constant patient population. The Fisher exact test was then used to recalculate the two‐sided *p*‐value, while iteratively adding of events until the *p*‐value reached or exceeded 0.05. The number of additional events required to reach *p* ≥ 0.05 was defined as the FI. Computations were carried out using RevMan 5.3.

## RESULTS

### Eligible studies

Seven studies [8, 9, 24, 25, 26, 27, 28] containing data comparing small bites versus large bites in the closure of midline laparotomies were included (Table 1). The initial search found 1433 studies. After excluding duplicates and unrelated studies based on abstract triage, 28 full texts were assessed for eligibility, out of which seven matched the inclusion criteria and were systematically reviewed. The year of publication of included studies ranged from 2009 to 2024. The total number of included patients was 3807, split into two groups: SG (*n* = 1768) and control group (CG; *n* = 2039). In the SG the fascia was closed adhering to the small‐bites technique by using 5 mm bites of continuous 2–0 PDS or Monomax® every 5 mm. Only fascia was taken in the bites, without rectus muscle or subcutaneous fat. One study [26] used the same suture type in both groups (1 PDS, 5/5 mm in the SG and 10/10 mm in the CG). In the CG the fascia was closed in a standard mass closure or fascia‐only fashion with 1 or 0 PDS. Closure in the CG was at the surgeon's preference in two studies [27, 28] in which the CG was made of a historical cohort, before adopting the small‐bites technique (Table 2). A total of 787 (20.6%) procedures were emergency procedures. From the final cohort, 12.5% (*n* = 478) of patients had a previous laparotomy and 67.9% (*n* = 2097) underwent a gastrointestinal procedure. The mean age in the SG was 64.7 years versus 63 years in the CG. The mean BMI was 25.5 kg/m^2^ in both groups. The meta‐analysis included three RCTs [8, 9, 26], one controlled clinical trial (CCT) [25] and three case–control studies [24, 27, 28] (Table 1). Follow‐up spanned from 12 to 52 months (Table 2).

**TABLE 1:** Overview of included studies.

Author	Year	Type	SG (*n*)	CG (*n*)	SG suture	CG suture	SG closure technique	CG closure technique
Albertsmeier (ESTOIH) [8]	2022	RCT	215	210	2/0 Monomax®	1 Monomax®	5/5 mm, fascia only	10/10 mm, fascia only
Deerenberg (STITCH) [9]	2015	RCT	276	284	2/0 PDS	1‐loop PDS	5/5 mm, fascia only	10/10 mm, mass closure
deVries [24]	2019	Case–control	136	191	2/0 PDS	1‐loop PDS	5/5 mm, fascia only	10/10 mm, mass closure
Millbourn [25]	2009	CCT	356	381	2/0 PDS	1 PDS	5/5 mm, fascia only	10/10 mm, mass closure
Ozcan [26]	2024	RCT	87	86	1 PDS	1 PDS	5/5 mm, fascia only	10/10 mm, fascia only
Söderbäck [27]	2022	Case–control	518	602	2/0 PDS	Surgeon preference (most 0‐loop PDS)	5/5 mm, fascia only	Surgeon preference
Thorup [28]	2019	case control	180	285	2/0 PDS	Surgeon preference	5/5 mm, fascia only	Surgeon preference

Abbreviations: CCT, controlled clinical trial; CG, control group; NOS, Newcastle–Ottawa Scale; RCT, randomized controlled trial; SG, study group.

**TABLE 2:** Characteristics of included studies.

Author	Year	Type of surgery	Follow‐up (months)	Outcomes (statistically significant)			Fragility index comparison		
				SSI	WD	IH	SSI	WD	IH
Albertsmeier (ESTOIH) [8]	2022	Elective	36	Similar	Less in SG	NR	0	0	0
Deerenberg (STITCH) [9]	2015	Elective	12	Similar	Similar	Less in SG	0	0	3
deVries [24]	2019	Elective and emergency	22.7	Less in SG	Similar	Less in SG	NA	NA	NA
Millbourn [25]	2009	Elective and emergency	12	Less in SG	Similar	Less in SG	3	0	16
Ozcan [26]	2024	Elective	24	Less in SG	NR	Less in SG	0	NA	8
Söderbäck [27]	2022	Elective and emergency	36	Similar	Similar	Similar	NA	NA	NA
Thorup [28]	2019	Emergency	52	Similar	Similar	Less in SG	NA	NA	NA

Abbreviations: CG, control group (e.g., large bites); IH, incisional hernia; NR, not recorded; SG, study group (e.g. small bites); SSI, surgical site infection; WD, wound dehiscence.

### Demographics and risk factors

#### Male gender

**FIGURE 3:**
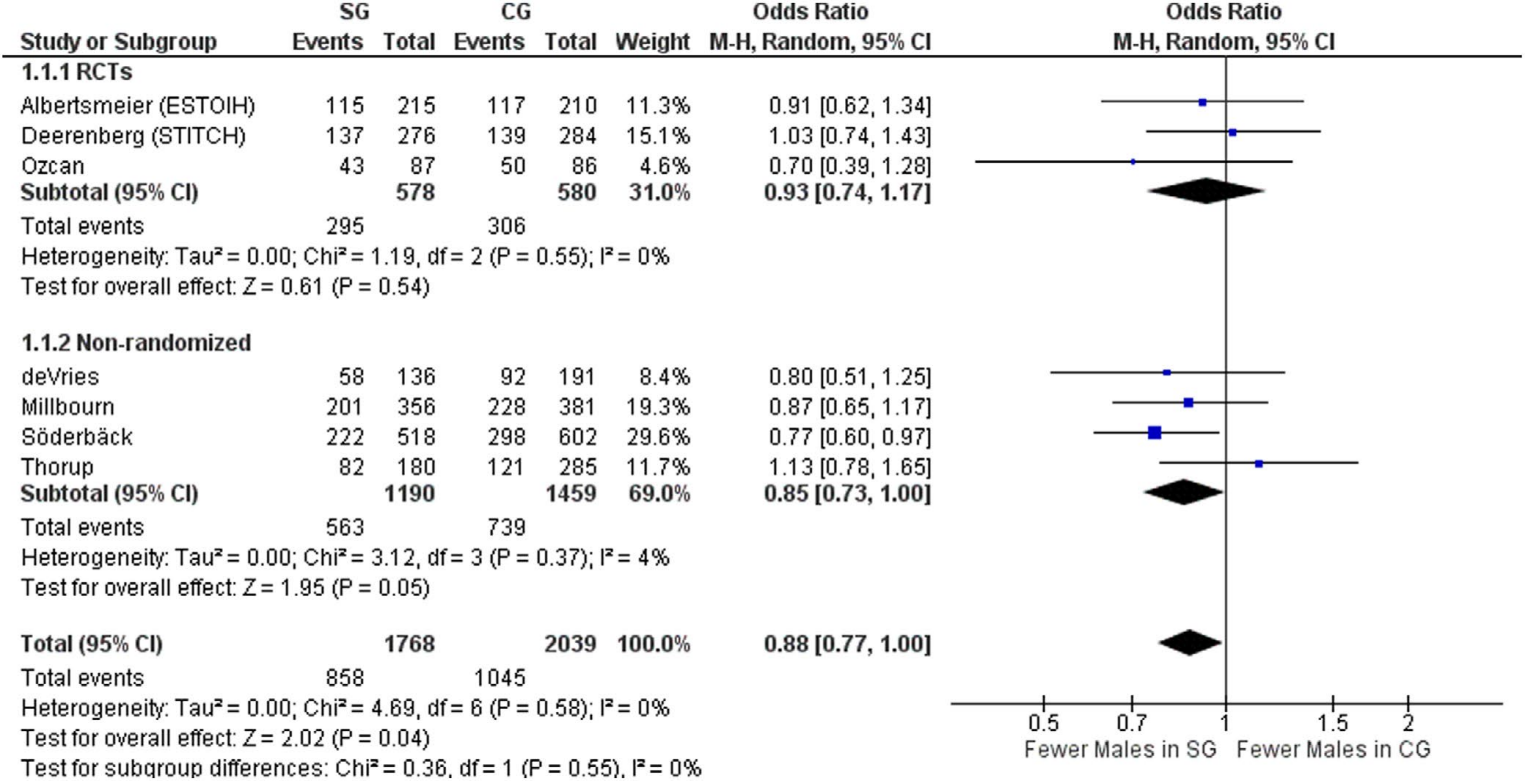
Study group (SG) versus control group (CG) and number of male patients (M‐H, Mantel‐Haenszel test; RCTs, randomized controlled trials).

All studies describing 3807 patients included data on the gender of patients. Overall, there were fewer men in the SG (OR 0.88, 95% CI 0.77–1.00, *p* = 0.04, χ^2^ = 4.69, *I* ^2^ = 0%) due to the significantly lower number of male patients included in the SG in the nonrandomized studies. When considering only RCTs, both groups were similar with low interstudy heterogeneity (OR 0.93, 95% CI 0.74–1.17, *p* = 0.54, χ^2^ = 1.19, *I* ^2^ = 0%; Figure 3).

#### Smoking

**FIGURE 4:**
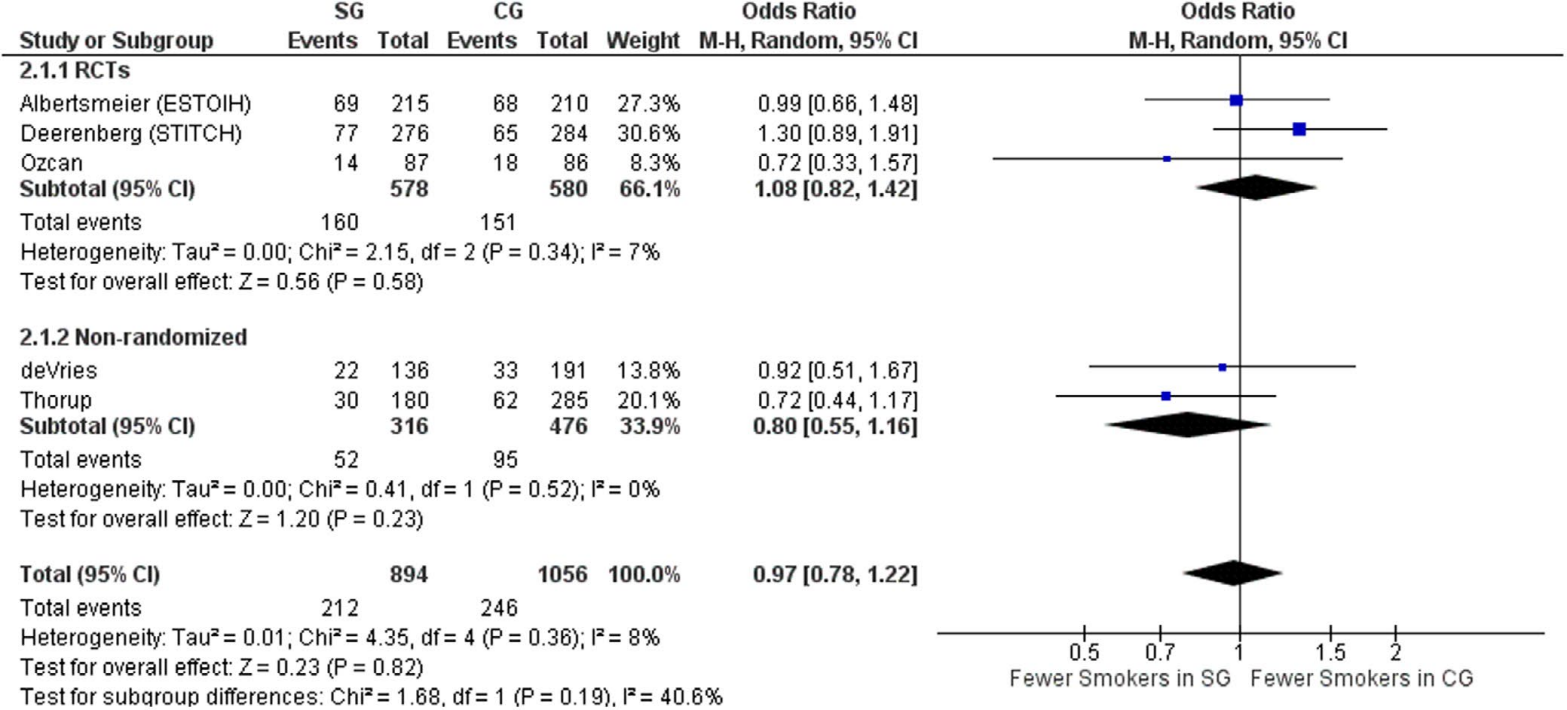
Study group (SG) versus control group (CG) and number of smokers (M‐H, Mantel‐Haenszel test; RCTs, randomized controlled trials).

Five studies, three RCTs [8, 9, 26] and two nonrandomized [24, 28] including data on 1950 patients, described how many smokers were in each group. There was no significant difference in number of smokers between the two groups, with low interstudy heterogeneity (OR 0.97, 95% CI 0.78–1.22, *p* = 0.82, χ^2^ = 4.35, *I* ^2^ = 8%; Figure 4).

#### ASA grades 3 and 4

**FIGURE 5:**
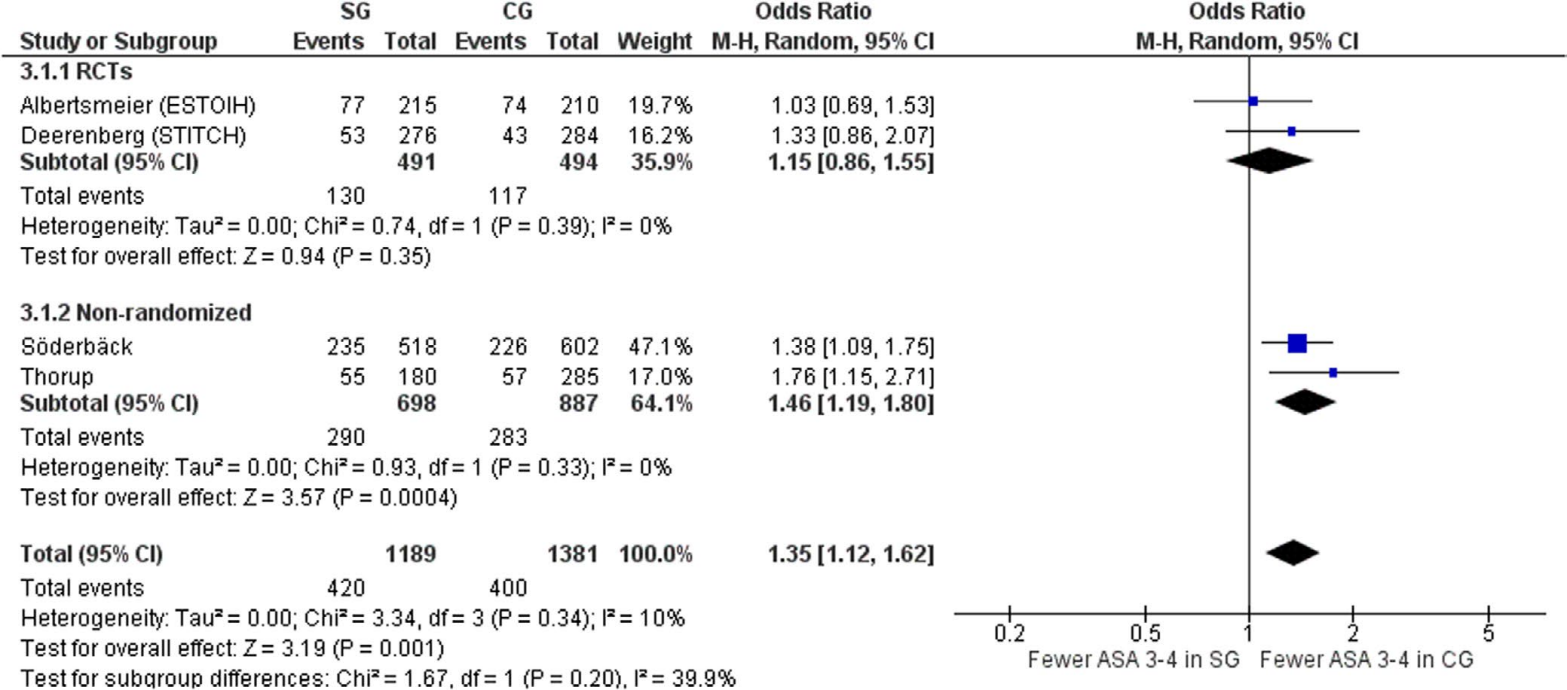
Study group (SG) versus control group (CG) and number of American Society of Anesthesiologists (ASA) grade 3–4 patients (M‐H, Mantel‐Haenszel test; RCTs, randomized controlled trials).

Four studies, two RCTs [8, 9] and two nonrandomized [27, 28] including data on 2570 patients, mentioned ASA grade in the two groups. Overall, there were significantly more ASA grade 3 and 4 patients in the SG (OR 1.35, 95% CI 1.12–1.62, *p* = 0.001, χ^2^ = 3.34, *I* ^2^ = 10%) due to the significantly higher number of ASA 3–4 patients in the SG in the nonrandomized studies. When considering RCTs only, both groups were similar, with low interstudy heterogeneity (OR 1.15, 95% CI 0.86–1.55, *p* = 0.35, χ^2^ = 0.74, *I* ^2^ = 0%) (Figure 5).

#### Emergency surgery

**FIGURE 6:**
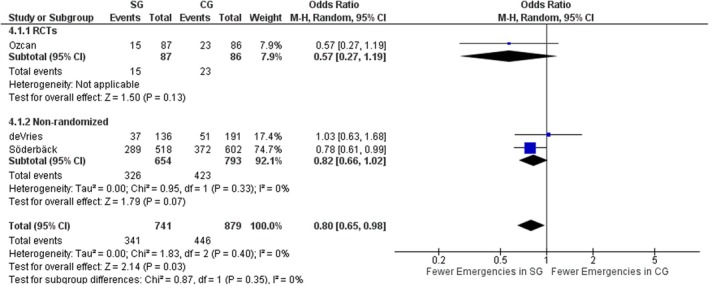
Study group (SG) versus control group (CG) and rate of emergency procedures (M‐H, Mantel‐Haenszel test; RCTs, randomized controlled trials).

Three studies, one RCT [26] and two nonrandomized [24, 27] including data on 787 patients, reported on the number of emergency operations performed in each group. Overall, there were significantly fewer emergency procedures performed in the SG (OR 0.80, 95% CI 0.65–0.98, *p* = 0.03, χ^2^ = 1.83, *I* ^2^ = 0%) based on the nonrandomized studies, while in the RCT both groups were similar (Figure 6).

#### Previous laparotomy

**FIGURE 7:**
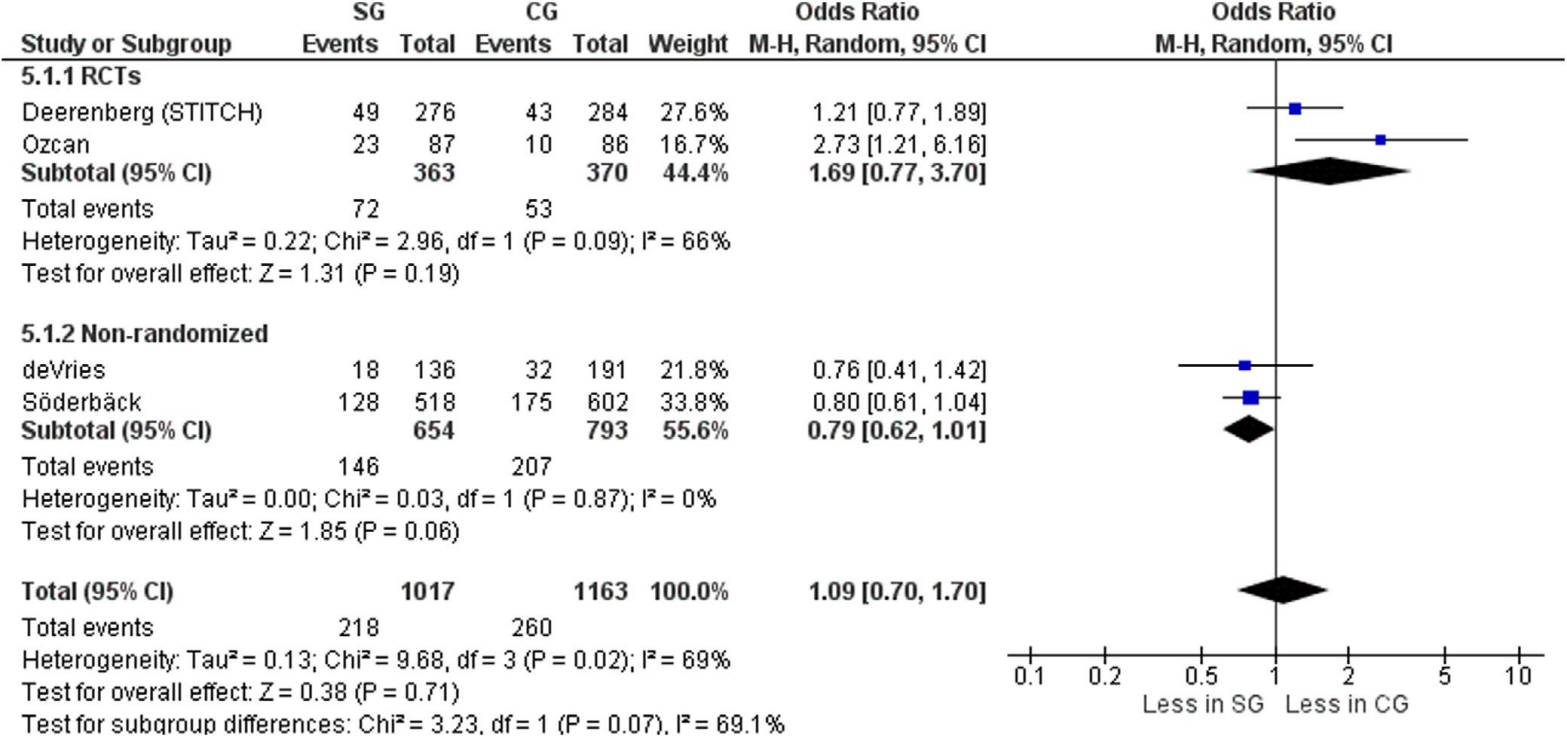
Study group (SG) versus control group (CG) and number of patients with previous laparotomies (M‐H, Mantel‐Haenszel test; RCTs, randomized controlled trials).

Four studies, two RCTs [9, 26] and two nonrandomized [24, 27] including data on 478 patients, provided data on the number of patients with previous midline surgery in the two groups as a risk factor for IH. There was no significant difference between SG and CG (OR 1.09, 95% CI 0.70–1.70, *p* = 0.71, χ^2^ = 9.68, *I* ^2^ = 69%) (Figure 7).

#### Gastrointestinal procedures

**FIGURE 8:**
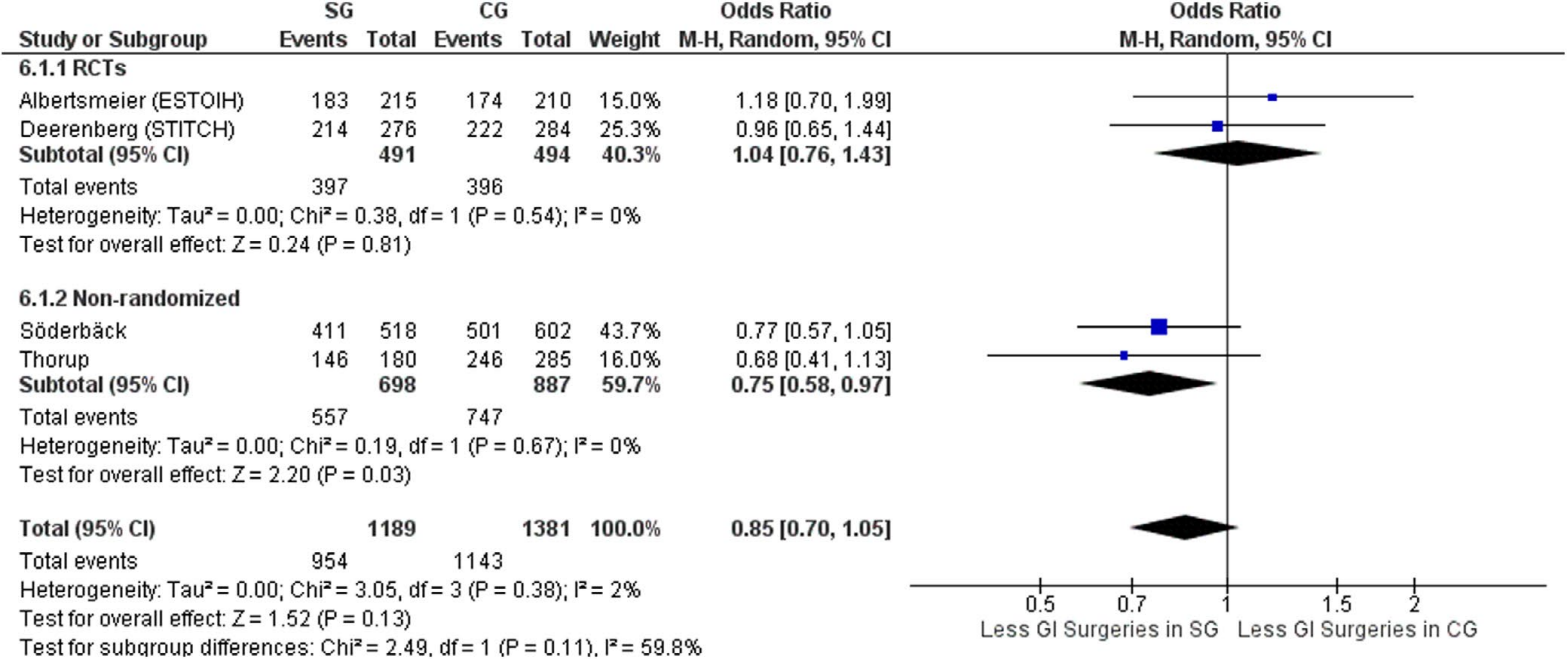
Study group (SG) versus control group (CG) and rate of gastrointestinal (GI) procedures (M‐H, Mantel‐Haenszel test; RCTs, randomized controlled trials).

Four studies, two RCTs [8, 9] and two nonrandomized [27, 28] including data on 2097 patients, compared the two groups in terms of type of operation. Gastrointestinal procedures were considered as high‐risk operations for development of SSI and IH. Overall, there were similar rates of gastrointestinal procedures performed in both groups (OR 0.85, 95% CI 0.70–1.05, *p* = 0.13, χ^2^ = 3.05, *I* ^2^ = 2%); however, when considering only nonrandomized studies the number of gastrointestinal procedures was less in the SG (OR 0.75, 95% CI 0.58–0.97, *p* = 0.03, χ^2^ = 0.19, *I* ^2^ = 0%) (Figure 8), whereas in the RCTs both groups had similar rates of gastrointestinal surgery.

### Intraoperative outcomes

#### Wound length

**FIGURE 9:**
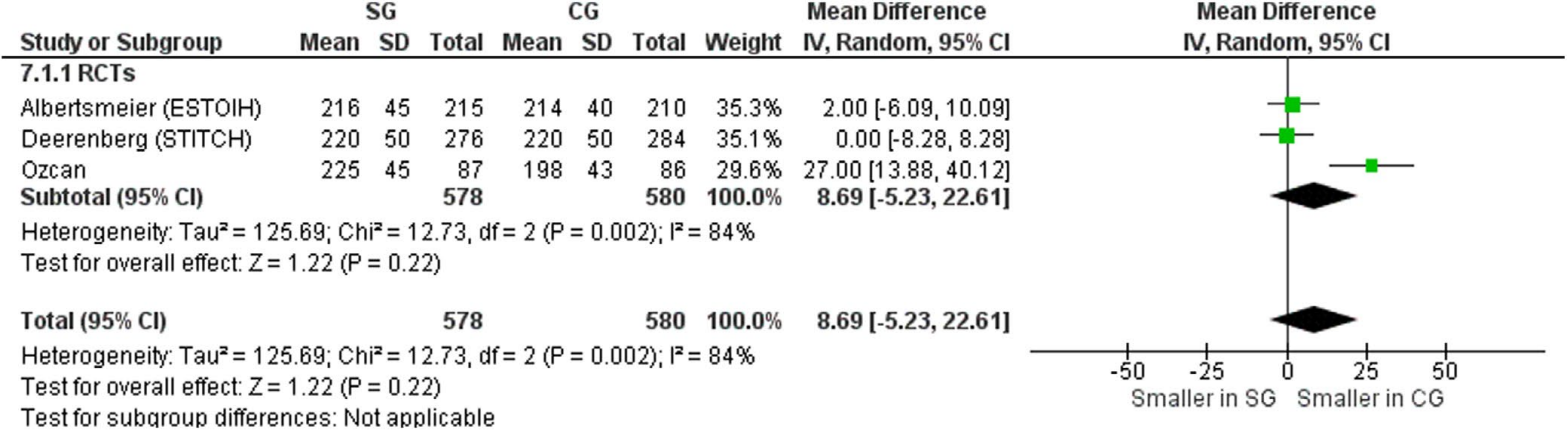
Study group (SG) versus control group (CG) and fascial incision length (M‐H, Mantel‐Haenszel test; RCTs, randomized controlled trials).

Three RCTs [8, 9, 26] including data on 1158 patients were analysed based on fascial incision length between the SG and CG. There was no significant difference in terms of wound length (mean difference 8.69 mm, 95% CI 5.23–22.61, *p* = 0.22, χ^2^ = 12.73, *I* ^2^ = 84%) (Figure 9).

#### Closure time

**FIGURE 10:**
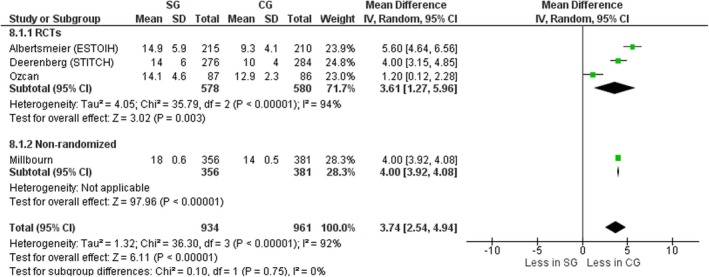
Study group (SG) versus control group (CG) and closure time (M‐H, Mantel‐Haenszel test; RCTs, randomized controlled trials).

Four studies, three RCTs [8, 9, 26] and one nonrandomized [25] including data on 1895 patients, measured the time required for fascial closure between small bites and large bites. Time for closure was significantly longer in the SG, with a mean difference of 3.74 min (95% CI 2.54–4.94, *p* < 0.00001, χ^2^ = 36.30, *I* ^2^ = 92%) (Figure 10).

#### Suture length

**FIGURE 11:**
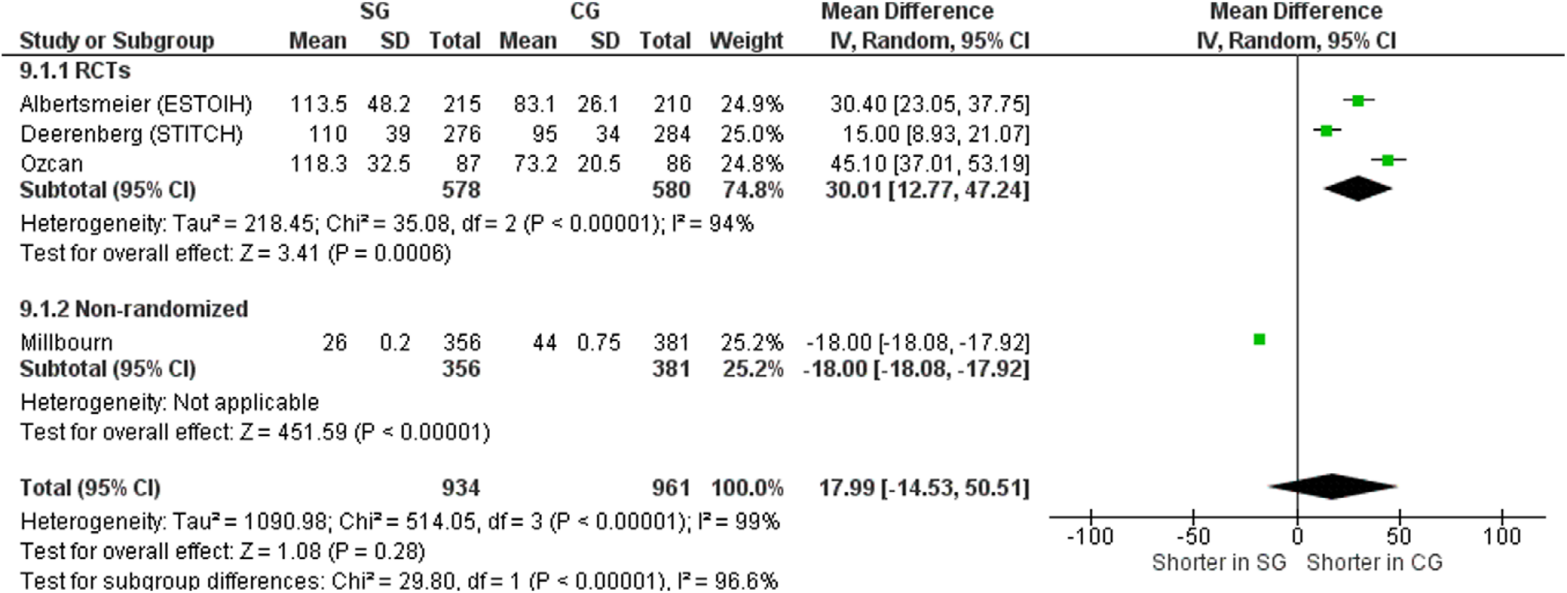
Study group (SG) versus control group (CG) and suture length (M‐H, Mantel‐Haenszel test; RCTs, randomized controlled trials).

Four studies, three RCTs [8, 9, 26] and one nonrandomized [25] including data on 1895 patients, compared the total suture length between the two techniques. Suture length was overall shorter in the large‐bites group with a mean difference of 17.99 cm (95% CI 14.53–50.51, *p* < 0.00001, χ^2^ = 514.05, *I* ^2^ = 99%). Millbourn et al. [25] reported a shorter suture length in the SG (26 mm vs. 44 mm) (Figure 11).

#### Suture to wound length ratio

**FIGURE 12:**
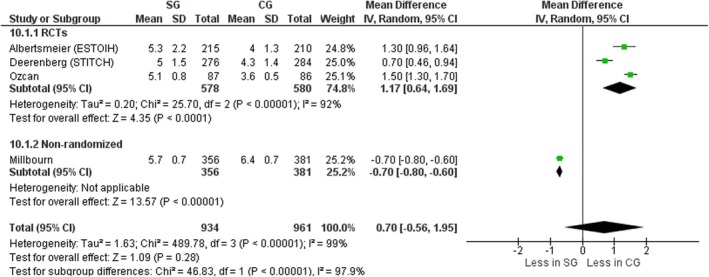
Study group (SG) versus control group (CG) and suture to wound length ratio (M‐H, Mantel‐Haenszel test; RCTs, randomized controlled trials).

Four studies, three RCTs [8, 9, 26] and one nonrandomized [25] including data on 1895 patients, compared the ratio between suture length and wound length between the two groups. The ratio was significantly smaller in the large‐bites group, suggesting that longer sutures were used in the small‐bites group reported to the wound length (mean difference 0.70, 95% CI 0.56–1.95, *p* < 0.00001, χ^2^ = 489.78, *I* ^2^ = 99%). Interestingly, Millbourn et al. [25] reported a smaller suture to wound length ratio in the SG (5.7 vs. 6.4) (Figure 12).

### Postoperative outcomes

#### Surgical site infections

**FIGURE 13:**
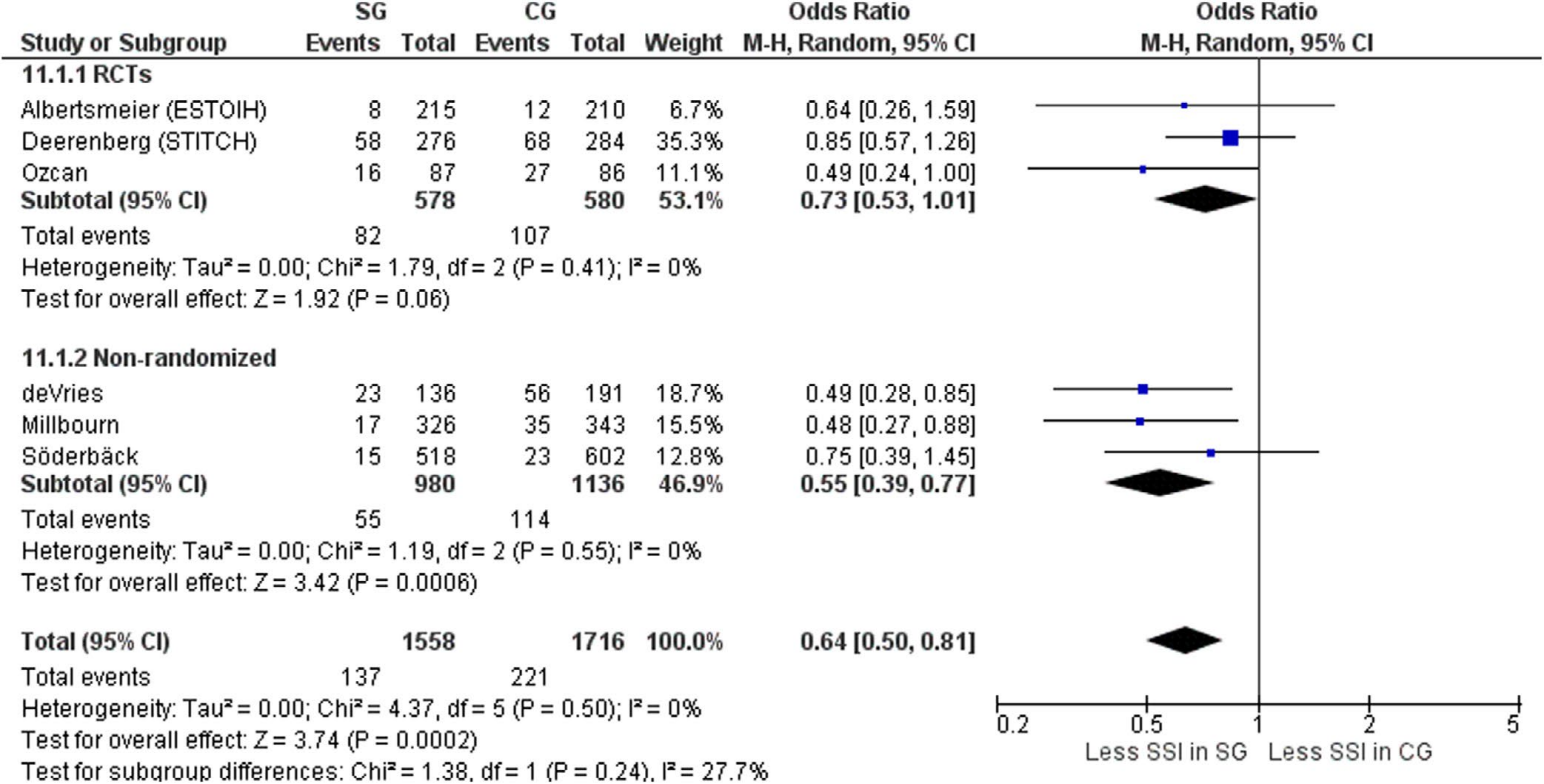
Study group (SG) versus control group (CG) and surgical site infection (SSI) (M‐H, Mantel‐Haenszel test; RCTs, randomized controlled trials).

Six studies, three RCTs [8, 9, 26] and three nonrandomized [24, 25, 27] including data on 3274 patients, compared rates of SSI between patients with small‐bites and patients with large‐bites abdominal closure. Overall, significantly fewer wound infections (superficial and deep) occurred in the small‐bites group, without significant interstudy heterogeneity (OR 0.64, 95% CI 0.50–0.81, *p* = 0.0002, χ^2^ = 4.37, *I* ^2^ = 0%) (Figure 13). When considering only RCTs, the difference did not reach significance (OR 0.73, 95% CI 0.53–1.01, *p* = 0.06, χ^2^ = 1.79, *I* ^2^ = 0%) (Figure 13). Note that the FI was 0 for all three RCTs, indicating a lack of robustness (Table 2).

#### Wound dehiscence

**FIGURE 14:**
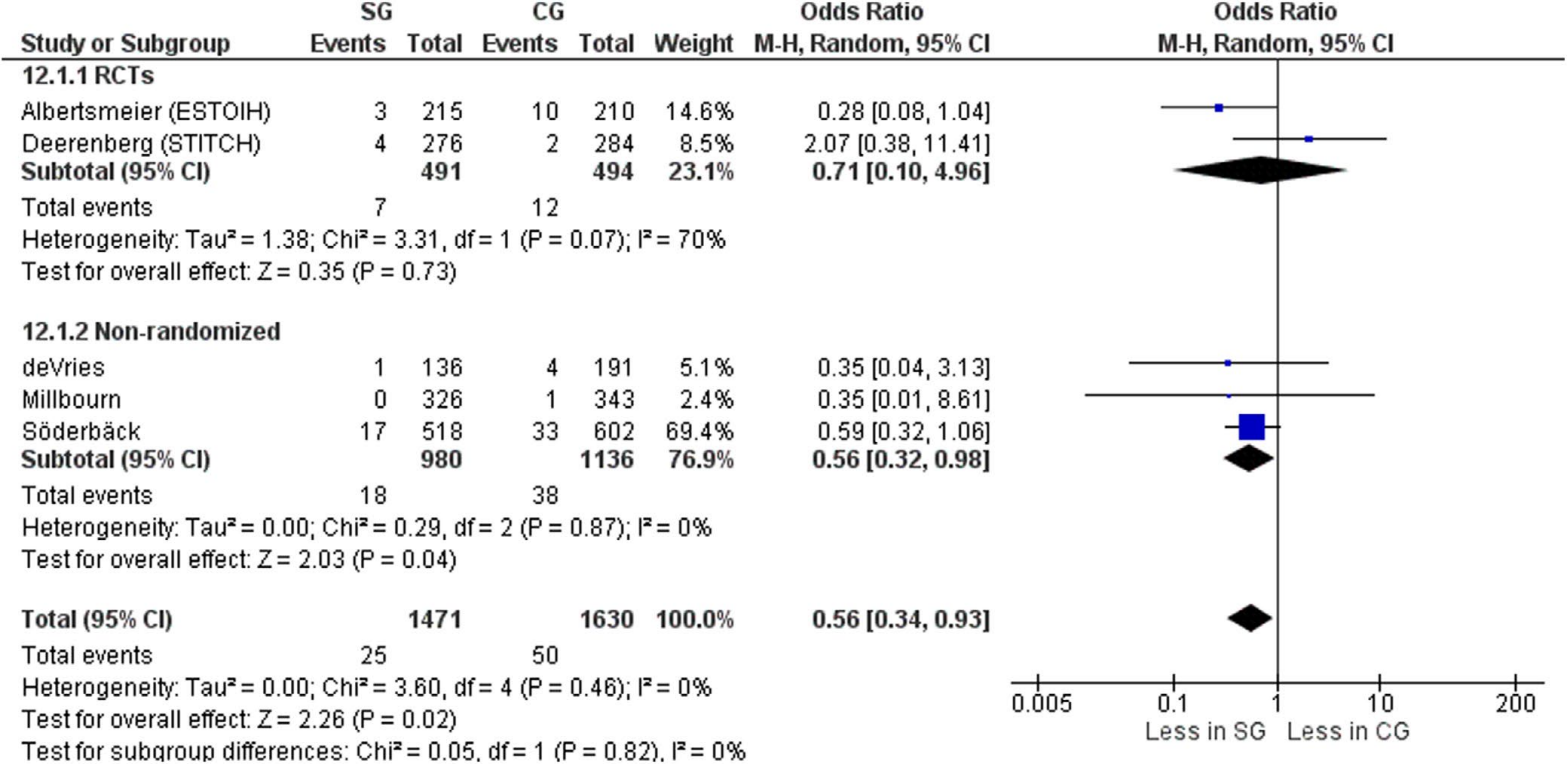
Study group (SG) versus control group (CG)and wound dehiscence (M‐H, Mantel‐Haenszel test; RCTs, randomized controlled trials).

Five studies, two RCTs [8, 9] and three nonrandomized [24, 25, 27] including data on 3101 patients, compared rates of wound dehiscence, both superficial and total dehiscence (burst abdomen). Overall, significantly less wound dehiscence occurred in the small‐bites group, without significant interstudy heterogeneity (OR 0.56, 95% CI 0.34–0.93, *p* = 0.02, χ^2^ = 3.60, *I* ^2^ = 0%) driven by results from nonrandomized studies (Figure 14). When considering RCTs only, the wound dehiscence rate was similar between the two groups (OR 0.71, 95% CI 0.10–4.96, *p* = 0.73, χ^2^ = 3.31, *I* ^2^ = 70%) with a FI of 0, emphasizing insignificance (Table 2).

#### Incisional hernia

**FIGURE 15:**
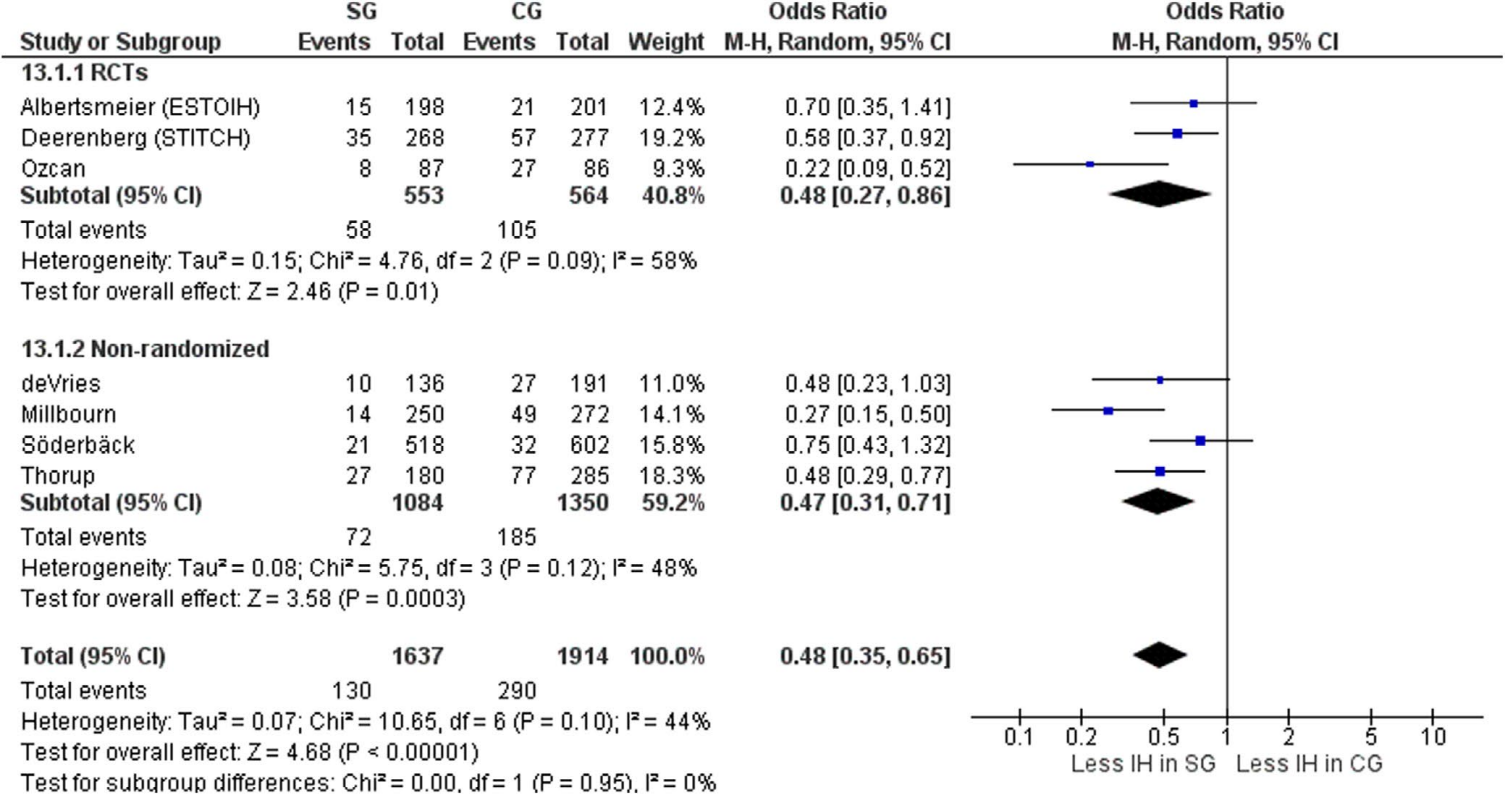
Study group (SG) versus control group (CG) and incisional hernia (IH) (M‐H, Mantel‐Haenszel test; RCTs, randomized controlled trials).

All studies describing 3551 patients analysed the incidence of IH between the two groups. Significantly fewer IHs occurred in the small‐bites group compared with the large‐bites group (OR 0.48, 95% CI 0.35–0.65, *p* < 0.00001, χ^2^ = 10.65, *I* ^2^ = 44%) (Figure 15). When considering RCTs only the results remained similar; however, the significant difference in favour of small bites is dependent on the STITCH trial results having a FI of 3, meaning that if three patients in the large‐bites group were not to have developed IH the results favouring small bites would have become unsignificant. The only acceptable FI of 8 was found in the Ozcan et al. [26] trial, which did favour small bites in terms of the IH rate (Table 2).

## DISCUSSION

Overall, small bites is associated with lower rates of SSI and IH when a meta‐analysis is performed of existing evidence combining retrospective and randomized data. The evidence supporting this is only significant for IH rates when examining RCTs, with fragility evident among existing randomized studies highlighting a requirement for further detailed data in this area.

To our knowledge this is the first meta‐analysis to compare surgical outcomes of small‐bites technique versus the standard large‐bites technique in terms of patient risk factors and incidence of SSI, wound dehiscence and IH with an examination of the fragility of randomized studies. At a glance the available literature supports the adoption of small bites, especially considering the latest European Hernia Society guidelines; however, the data are highly heterogenous. On one hand we have a group of retrospective case–control studies, some included here, that favour small bites, but we found significant differences in terms of patient selection, such as fewer men or fewer emergency procedures in the SG. These weaken the evidence due to the high risk of selection bias. More so, the studies were designed to compare a standardized technique of closing the abdomen, through small‐bites adoption, versus a nonstandardized historical way, based on surgeon preference. This implies another important risk of selection and performance bias, because in the CG the closure could have been performed in various ways. Thus, the favourable results in the SG may be attributed to the standardized and thorough way of closing the abdomen rather than the suture or technique itself. On the other hand, we have the evidence provided by RCTs, namely the STITCH trial [9], the ESTOIH trial [8] and a newly published trial from Ozcan et al. [26]. The trial published by Millbourn et al. [25] in 2009 was considered a CCT due to the weak randomization method (by alternating weeks). When analysing only RCTs, the preoperative risk factors were all similar between the two groups. Also, in all RCTs the operative variables were measured, emphasizing more robust evidence as in all patients the wound length was measured, closure was done similarly in both groups and sutures were measured in both groups confirming adherence to the allocated technique. Interestingly, in Millbourn et al. [25] despite using a 5/5 mm closure in the SG versus a 10/10 mm one in the CG, the suture length and suture to wound length ratio were smaller for the small‐bites technique, which is difficult to explain for a technique where more bites and less suture are used.

Published in 2015, the double‐blinded, multicentre STITCH trial [9] was the first RCT to show fewer IHs after small bites at 1 year of follow‐up and with similar SSI and wound dehiscence rates when compared with the large‐bites technique; however, the robustness of evidence stands on a FI of 3, meaning that if three patients were to have had a different outcome the results favouring small bites in terms of the IH rate would become insignificant. The ESTOIH trial [8] is the other double‐blinded, multicentre RCT that initially showed a trend favouring small bites at 30 days and 1 year in terms of wound dehiscence and IH. However, the updated results at 36 months of follow‐up showed similar IH rates. Although the results of the above trials proved the safety of small bites, adoption among colorectal surgeons is still poor. One view is that the above trials included only elective cases and a significant number of noncolorectal surgeries (i.e. gynaecological, miscellaneous). The recently published trial from Ozcan et al. [26], which included only colorectal cases (both elective and emergency operations, although it did exclude emergencies for perforated bowel, which is a bias), showed less SSI in the small‐bites groups and a lower IH rate after 24 months of follow‐up with an acceptable FI of 8. Noteably, in both groups a similar type of suture was used (no. 1 PDS), thus the authors deviated from the standard small‐bites technique with 2/0 PDS. Also, the single‐centre design lacks external validation and the outcome measurement was done by the same team that performed closure, which implies a high risk of bias in measurement and reporting.

Further research should clarify certain issues. In most cases the small‐bites technique was compared with a large‐bite mass closure technique. It is unclear whether the difference in outcomes is related to the distance between bites, to the type of suture used or to the difference in tissue thickness taken into the bites. It is known from experimental studies [6, 29, 30, 31] that loading muscle and subcutaneous fat into the bite will only lead to tissue necrosis. Similarly, loop PDS has been shown to be more traumatic, increasing the risk of buttonhole hernias, and larger bites may lead to tissue constriction and ischaemia, ultimately allowing separation of the fascial edges. Ensuring bites are taken every 5 mm and only the anterior fascia is loaded in the sutures may lead to similar outcomes even when using a thicker suture, such as 0 or 1 PDS and we have the results from Ozcan et al. [26] and the ESTOIH trial [8] to support this.

There are limitations to our study. We chose to include retrospective studies, not only RCTs, as comparing small bites with past cohorts may not be influenced by performance or study bias and could reflect real‐word clinical practice, albeit with important risks of selections bias. Despite our efforts to compare the two groups in terms of risk factors for SSI and IH, not all studies provided equally distributed data in this regard. Not all risk factors for IH development such as chronic obstructive pulmonary disease and steroid use could be fully defined for each group. Based on this meta‐analysis, the use of the small‐bites technique appears to be safe and noninferior to standard closure, with some conflicting evidence based on RCTs regarding the improvement in IH rates over time.

## CONCLUSION

Small bites is not inferior to large bites. Rates of IH may be lower after small‐bites closure, but RCTs have differing results with fragility evident in some reporting favourably for small bites. Further research is required before widespread adoption of small bites as the new standard technique for abdominal closure.

## AUTHOR CONTRIBUTIONS

**Stefan Morarasu:** Conceptualization; writing – original draft; methodology; writing – review and editing; software; formal analysis; data curation. **Sorinel Lunca:** Conceptualization; writing – original draft; methodology; writing – review and editing; supervision. **Luke O'Brien:** Data curation; software; methodology; writing – review and editing. **Paul Lynch:** Writing – review and editing; methodology; data curation; formal analysis. **Ana Maria Musina:** Writing – review and editing; methodology; formal analysis; data curation. **Cristian Ene Roata:** Software; data curation; writing – review and editing; methodology. **Raluca Zaharia:** Methodology; data curation; writing – review and editing. **Wee Liam Ong:** Validation; methodology; writing – review and editing; data curation. **Gabriel‐Mihail Dimofte:** Supervision; data curation; formal analysis; writing – review and editing. **Cillian Clancy:** Writing – review and editing; writing – original draft; supervision; data curation; formal analysis; software; project administration; methodology; conceptualization; investigation; funding acquisition.

## FUNDING INFORMATION

No funding or financial assistance was received by the authors.

## CONFLICT OF INTEREST STATEMENT

The authors have no conflicts of interest to disclose.

## ETHICS STATEMENT

No ethical approval was required for this study as it is a meta‐analysis. No individual patient data was used.

## Data Availability

The data that support the findings of this study are available from the corresponding author upon reasonable request.
